# Increase in Low-Frequency Oscillations in fNIRS as Cerebral Response to Auditory Stimulation with Familiar Music

**DOI:** 10.3390/brainsci12010042

**Published:** 2021-12-29

**Authors:** Giulio Bicciato, Emanuela Keller, Martin Wolf, Giovanna Brandi, Sven Schulthess, Susanne Gabriele Friedl, Jan Folkard Willms, Gagan Narula

**Affiliations:** 1 Neurocritical Care Unit, Department of Neurosurgery, Institute of Intensive Care Medicine, University Hospital, University of Zurich, 8091 Zürich, Switzerland; emanuela.keller@usz.ch (E.K.); giovanna.brandi@usz.ch (G.B.); schulthess.sven@gmail.com (S.S.); SusanneGabriele.friedl@usz.ch (S.G.F.); janfolkard.willms@usz.ch (J.F.W.); gnarula1986@gmail.com (G.N.); 2 Department of Neurology, University Hospital, University of Zurich, 8091 Zürich, Switzerland; 3 Biomedical Optics Research Laboratory, Department of Neonatology, University Hospital, University of Zurich, 8091 Zürich, Switzerland; martin.wolf@usz.ch

**Keywords:** fNIRS, music, low-frequency oscillations, LFO, consciousness

## Abstract

Recognition of typical patterns of brain response to external stimuli using near-infrared spectroscopy (fNIRS) may become a gateway to detecting covert consciousness in clinically unresponsive patients. This is the first fNIRS study on the cortical hemodynamic response to favorite music using a frequency domain approach. The aim of this study was to identify a possible marker of cognitive response in healthy subjects by investigating variations in the oscillatory signal of fNIRS in the spectral regions of low-frequency (LFO) and very-low-frequency oscillations (VLFO). The experiment consisted of two periods of exposure to preferred music, preceded and followed by a resting phase. Spectral power in the LFO region increased in all the subjects after the first exposure to music and decreased again in the subsequent resting phase. After the second music exposure, the increase in LFO spectral power was less distinct. Changes in LFO spectral power were more after first music exposure and the repetition-related habituation effect strongly suggest a cerebral origin of the fNIRS signal. Recognition of typical patterns of brain response to specific environmental stimulation is a required step for the concrete validation of a fNIRS-based diagnostic tool.

## 1. Introduction

Perception of preferred music has been shown to simultaneously activate different cerebral regions and networks involved in consciousness, language, emotion, and memory processing [1,2]. Music therapy is thought to enhance neuroplasticity and to have a beneficial effect on the cognitive abilities of patients with consciousness disorders [3]. Moreover, auditory stimulation with preferred music is particularly sensitive for identifying awareness and has been shown to elicit a cortical response in minimally conscious state patients [4]. Measurement of brain response to preferred music may be used to detect covert consciousness in clinically unresponsive patients. Current available diagnostic tools to measure cortical activity, such as electroencephalography (EEG), acoustic evoked potentials, positron emission tomography, and functional magnetic resonance imaging, present several limitations in terms of accuracy, logistics, and costs [5,6,7]. Functional near-infrared spectroscopy (fNIRS) could represent a promising non-invasive, bedside method to monitor cortical activity and eventually detect signs of consciousness in critically ill neurological patients [8,9,10]. Unfortunately, fNIRS is not yet available as a validated clinical tool. Indeed, there is no established methodology to interpret the hemodynamic information in a way that concretely supports clinical decisions. Hence, recognition of typical patterns of brain response to preferred music in healthy subjects is a required step to develop a tool that we can concretely use to assess clinically unresponsive patients and eventually detect covert consciousness.

Past fNIRS studies have shown that auditory stimulation induces an increase in cerebral oxygenation, measured in the temporal and frontal regions in newborns [11,12]. On the contrary, in adults listening to music while performing other cognitive tasks, oxygenation in the frontal areas showed a slight reduction [13,14]. Differently to high-frequency repetitive stimulation, during long stimulation (such as hearing music for several minutes), the standard time-course analysis of hemoglobin oxygenation is difficult to interpret as we do not know what to expect for every change of rhythm/tonality, etc., during the music piece. In our study, we propose an alternative approach to fNIRS analysis consisting of the investigation of the hemodynamic oscillations of the cerebral cortex by means of spectral analysis [15]. Analysis in the frequency domain has the practical advantage of simply eliminating some of the interference from systemic signals (e.g., heartbeat, respiration), by excluding their frequency bands, but it also has a deeper physiological significance. In fact, phylogenetically preserved oscillatory electric and hemodynamic networks of various size are thought to be crucial in the local and global transmission of information in the brain [16]. The main frequencies of hemodynamic oscillation in the cortex show task-related shifts during cognitive activation. Furthermore, synchronized frequency shifts in different cortical regions could underpin patterns of cortical connectivity [17,18]. In fact, based on functional MRI (fMRI) studies, oscillations in the blood-oxygen-level-dependent (BOLD) signal between ca. 0.01 and 0.1 Hz reflect coupled neuronal–hemodynamic activity [19]. In particular, there is a correspondence between specific cognitive functions and localized hemodynamic oscillations within characteristic frequencies [20,21]. Resting state networks described by fMRI rely on such hemodynamic low-frequency BOLD oscillations [22]. Analogously to fMRI, fNIRS also delivers information on cortical hemodynamics by measuring variations in the concentration of oxygenated hemoglobin (O2Hb) and deoxygenated hemoglobin (HHb) [23,24]. Within the low-frequency region of the spectrum, a further differentiation between low-frequency oscillations (LFO) centered around 0.1 Hz and very-low-frequency oscillations (VLFO) below 0.05 Hz has been described, even if the exact extent of each range is uncertain and its evaluation varies between authors [25,26,27]. VLFO are thought to be generated by a central pacemaker and may be involved in interhemispheric long-distance functional connection, although they are also influenced by metabolic activity in micro-vessels and changes in PaCO2 levels in the blood [26,28]. LFO partially reflect systemic Mayer waves but are also believed to reflect local vasomotion in terminal arterioles as part of local autoregulation, influenced by sympathetic nervous activity [29]. The amplitude of LFO is locally reduced after loss of autoregulation after acute ischemic stroke [30].

The aim of this pilot study was to seek specific variations in oscillatory signal in VLFO and LFO regions as a marker of the cognitive response to music and to further our knowledge about the physiological meaning of hemodynamic cerebral oscillations.

## 2. Materials and Methods

### 2.1. Participants

We enrolled a group of 6 healthy, right-handed volunteers (2 females, 4 males). Mean age was 41.2 years (±12.6). The study was approved by the local ethics committee. Written consent was given by every participant.

### 2.2. Experiment Design

**Figure 1:**
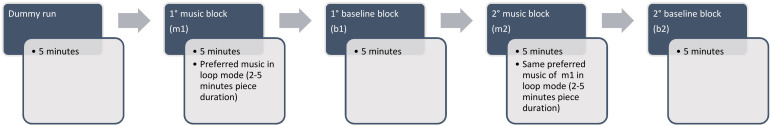
Illustration of the 5 subsequent blocks: baseline 1 (b1), music 1 (m1), baseline 2 (b2), music 2 (m2), baseline 3 (b3).

All the subjects were asked to listen to their favorite music. Subjects 1, 4, 5 chose pop/rock music with singing and melody, while subjects 2, 3, and 6 chose classical instrumental pieces. All pieces lasted between 2 and 5 min and were played in a loop. Music was played on a Samsung S8 mobile phone connected to disposable headphones; the volume level was always set under the security lock furnished by the device. Since sound perception depends both on the decibel level of the transmission and on the hearing ability of the subject, no standardized value could have been chosen. Every subject was preliminarily asked about the optimal volume level setting to make the music sufficiently audible while avoiding annoyance. None of the subjects enrolled had documented moderate or severe hearing loss. The experiment was performed in a noiseless environment, but no other sound attenuation methods were used because they would be difficult to reproduce in a clinical setting. The experiment consisted of several blocks, including an initial and a final period of resting state recording, separated by periods when subjects listened to music. The experiment consisted of 5 subsequent blocks: baseline 1 (b1), music 1 (m1), baseline 2 (b2), music 2 (m2), baseline 3 (b3). Each block lasted 5 min; between the subsequent blocks, there were no pauses or other interruptions of the protocol. The first baseline block was preceded by a first “dummy” NIRS measurement of 5 min for calibration to verify the good quality of the signal and exclude major artifacts (Figure 1).

### 2.3. NIRS Measurement

**Figure 2:**
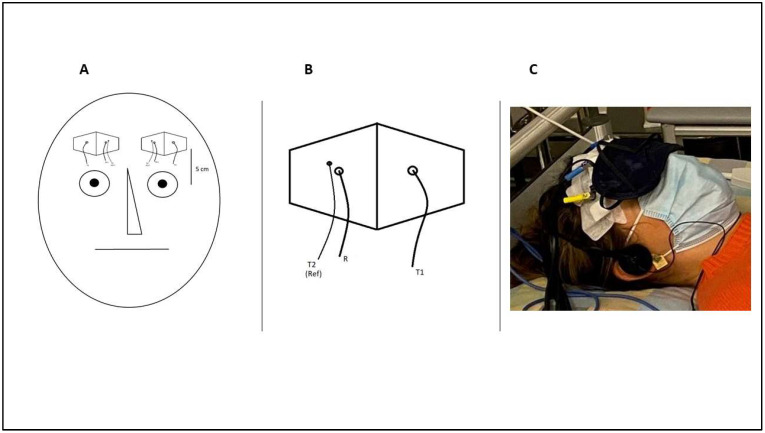
(**A**) The optodes were placed on the forehead approximately 5 cm above the mediopupillary line. (**B**) Optode configuration. R = receiver. T1 = main infrared light transmitter. T2 (reference) = secondary infrared light transmitter (“short channel”). (**C**) Subject 6 during experiment; the optodes were fixed with patches on the forehead.

Data were acquired and analyzed using OXYMON Mk III and Oxysoft (version 3.0, 103.3) (Manufacturer: Artinis Medical Systems B.V., Elst, The Netherlands). We performed near-infrared spectroscopy (NIRS), applying two optodes on the forehead bilaterally, approximately 5 cm above the mediopupillary line (Figure 2B). Each optode consisted of two light sources and one detector. Interoptode distance, i.e., the distance between the main light source and the light detector, was 35 mm. An auxiliary short channel had an interoptode distance of 10 mm (Figure 2A). The optical signal of the short channel was subtracted from the main signal to reduce the effect of the superficial signal originating from the scalp using the “subtract” function in Oxysoft software. The sampling frequency was set at 25 Hz. The differential pathlength factor (DPF) was set at 6. O2Hb and HHb were automatically computed according to the modified Lambert–Beer law using the Oxysoft software.

### 2.4. Systemic Vital Parameters

During the experiment, heart rate, oxygen saturation (SpO2), and respiratory rate were continuously monitored. All signals were acquired using a Philips Intellivue system (Philips Medical systems, Boeblingen, Germany) that ported data to a CNS Data Collector (Moberg ICU Solutions, Ambler, Philadelphia, PA, USA). We collected and exported the data from the CNS Data Collector from each bed using our proprietary IT system, ICU Cockpit. Signals were sampled at 1.024 Hz.

### 2.5. Processing of NIRS Data and Statistical Analysis

**Figure 3:**
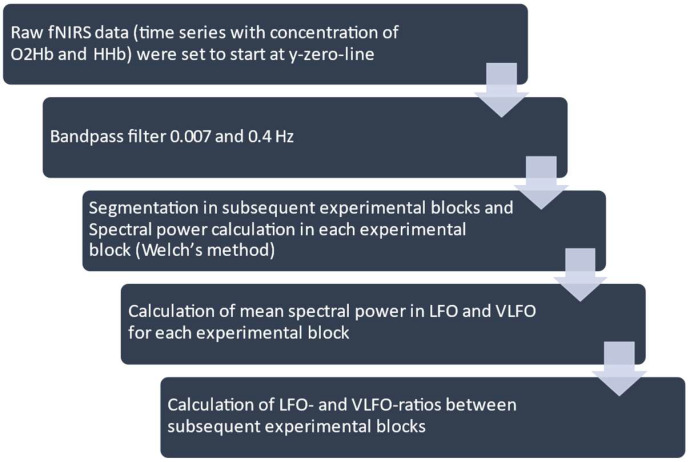
Processing of functional near-infrared spectroscopy (fNIRS) data. O2Hb = oxygenated hemoglobin. HHb = deoxygenated hemoglobin. LFO = low-frequency oscillations. VLFO = very-low-frequency oscillations.

**Figure A1:**
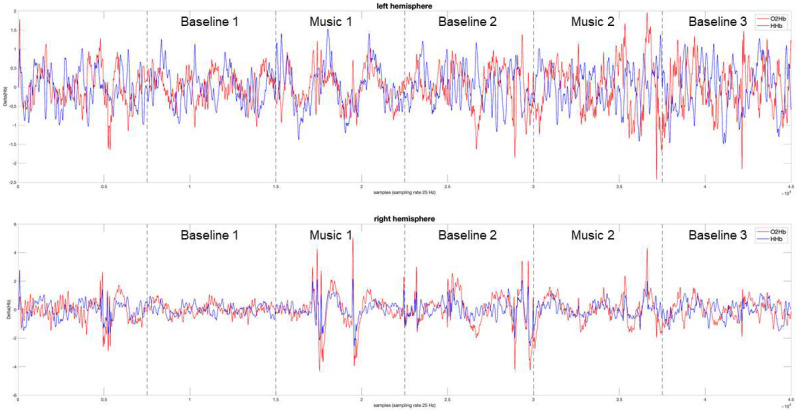
Example of time course fNIRS signal after the initial bandpass filter (0.007–0.4 Hz) for oxyhemoglobin (O2Hb) in red and deoxyhemoglobin (HHb) in blue, during the experiment in subject 6. On the *y*–axis is represented the variation (delta) in the concentration of O2Hb and HHb; on the *x*–axis are shown the samples (at sampling frequency of 25 Hz). Each experimental block is marked by a dashed line. At the top is demonstrated the signal on the left hemisphere; at the bottom is the signal on the right hemisphere.

Analyses were performed in MATLAB_R2019b (Mathworks, Natick, MA, USA). The raw fNIRS data, consisting of time series of the concentration of O2Hb and HHb, were obtained during the subsequent experimental blocks. The raw fNIRS data were first set to start at the y-zero line, and then they were bandpass-filtered between 0.007 and 0.4 Hz (filter order = 8, type = Butterworth). The higher limit of the bandpass filter was chosen to isolate cardiac and respiratory oscillations (a respiration frequency beyond 0.4 Hz, i.e., >24/min, would imply a pathological condition such as tachypnea or voluntary hyperventilation). The low-bandpass filter was applied to reduce slow-drift noise [31]. An example of a time course of fNIRS data after filtering is shown in the Appendix A (Figure A1). The signal was segmented into several blocks by the different conditions, b1, m1, b2, m2, and b3, according to the time points registered during the experiment. No major movement artifacts were found after inspection of the measured signals. Following analyses of fNIRS measurements were carried out for O2Hb and HHb in both hemispheres. For each block, we calculated the distribution of spectral power between 0.007 and 0.4 Hz according to Welch’s method. We used the MATLAB program *pwelch* with the following parameters: window length = 60 samples ∗ sampling frequency (sampling frequency = 25 Hz, i.e., the window length corresponds to the number of samples measured during 60 s); sample overlap = 30 samples ∗ sampling frequency (i.e., 50% overlap, standard setting of *pwelch* in Matlab); nfft (nonequispaced fast Fourier transform) = 2048 (standard nfft is the next power of to greater than the window length). According to the existing literature [25,26,31,32], a very low oscillatory range (very-low-frequency oscillation, VLFO) and a low oscillatory range (low-frequency oscillation, LFO) were defined, setting a cut-off of ≤0.04 and >0.04 Hz, respectively (Figure 3). To verify the robustness of the results, further analyses were performed using alternative cut-offs between LFO and VLFO, such as 0.03 and 0.05 Hz.

To evaluate the changes in the LFO and VLFO spectrum, we calculated the ratio between mean power in these two frequency ranges for different pairs of blocks of the experiment (e.g., from baseline to music); the ratio was then transformed with the natural logarithm (Ln) for the sake of a better graphical representation as follows:

$$\mathrm{LFO}.\mathrm{Ratiobaseline}\;\mathrm{to}\;\mathrm{music}=\mathrm{Ln}\left(\;\frac{\mathrm{mean}\left(\;\mathrm{LFO}.\mathrm{Powermusic}\;\right)}{\mathrm{mean}\left(\;\mathrm{LFO}.\mathrm{Powerbaseline}\;\right)}\;\right)$$

$$\mathrm{VLFO}.\mathrm{Ratiobaseline}\;\mathrm{to}\;\mathrm{music}=\mathrm{Ln}\left(\;\frac{\mathrm{mean}\left(\;\mathrm{VLFO}.\mathrm{Powermusic}\;\right)}{\mathrm{mean}\left(\;\mathrm{VLFO}.\mathrm{Powerbaseline}\;\right)}\;\right)$$

LFO.Ratio and VLFO.Ratio were thus calculated considering subsequent pairs of experimental blocks, i.e., the transition from baseline 1 (b1) to music 1 (m1), from m1 to baseline 2 (b2), from b2 to music 2 (m2), and from m2 to baseline 3 (b3), as follows:

$$\mathrm{LFO}.\mathrm{Ratiom}1/b1=\mathrm{Ln}\left(\;\frac{\mathrm{mean}\left(\;\mathrm{LFO}.\mathrm{Powermusic}1\;\right)}{\mathrm{mean}\left(\;\mathrm{LFO}.\mathrm{Powerbaseline}1\;\right)}\;\right)$$

$$\mathrm{LFO}.\mathrm{Ratiob}2/m1=\mathrm{Ln}\left(\;\frac{\mathrm{mean}\left(\;\mathrm{LFO}.\mathrm{Powerbaseline}2\;\right)}{\mathrm{mean}\left(\;\mathrm{LFO}.\mathrm{Powermusic}1\;\right)}\;\right)$$

$$\mathrm{LFO}.\mathrm{Ratiom}2/b2=\mathrm{Ln}\left(\;\frac{\mathrm{mean}\left(\;\mathrm{LFO}.\mathrm{Powermusic}2\;\right)}{\mathrm{mean}\left(\;\mathrm{LFO}.\mathrm{Powerbaseline}2\;\right)}\;\right)$$

$$\mathrm{LFO}.\mathrm{Ratiob}3/m2=\mathrm{Ln}\left(\;\frac{\mathrm{mean}\left(\;\mathrm{LFO}.\mathrm{Powerbaseline}3\;\right)}{\mathrm{mean}\left(\;\mathrm{LFO}.\mathrm{Powermusic}2\right)}\;\right)$$

$$\mathrm{VLFO}.\mathrm{Ratiom}1/b1=\mathrm{Ln}\left(\;\frac{\mathrm{mean}\left(\;\mathrm{VLFO}.\mathrm{Powermusic}1\;\right)}{\mathrm{mean}\left(\;\mathrm{VLFO}.\mathrm{Powerbaseline}1\;\right)}\;\right)$$

$$\mathrm{VLFO}.\mathrm{Ratiob}2/m1=\mathrm{Ln}\left(\;\frac{\mathrm{mean}\left(\;\mathrm{VLFO}.\mathrm{Powerbaseline}2\;\right)}{\mathrm{mean}\left(\;\mathrm{VLFO}.\mathrm{Powermusic}1\;\right)}\;\right)$$

$$\mathrm{VLFO}.\mathrm{Ratiom}2/b2=\mathrm{Ln}\left(\;\frac{\mathrm{mean}\left(\;\mathrm{VLFO}.\mathrm{Powermusic}2\;\right)}{\mathrm{mean}\left(\;\mathrm{VLFO}.\mathrm{Powerbaseline}2\;\right)}\;\right)$$

$$\mathrm{VLFO}.\mathrm{Ratiob}3/m2=\mathrm{Ln}\left(\;\frac{\mathrm{mean}\left(\;\mathrm{VLFO}.\mathrm{Powerbaseline}3\;\right)}{\mathrm{mean}\left(\;\mathrm{VLFO}.\mathrm{Powermusic}2\right)}\;\right)$$

The derived values of LFO.Ratio and VLFO.Ratio were used to evaluate the changes in the power spectrum as possible markers of changes in cortical activity among the different blocks of the experiment. These values were calculated separately for O2Hb and HHb. Changes in the LFO and VLFO spectrum were also assessed through effect size calculation. We calculated the effect size using both Cohen’s d (parametric) and Cliff’s delta (nonparametric). To Cohen’s d, a correction for the small sample size was applied, by multiplying the Cohen’s d for the following coefficient of correction (coefcor). Here, N refers to the number of subjects (N = 6). $\mu_{1}$ and $\mu_{2}$ represent the mean value (across the 6 subjects) of the average LFO or VLFO power within an experimental block (e.g., $\mu_{1}$ is average LFO in m1 and $\mu_{2}$ is average LFO in b1). SD is the pooled standard deviation, which is the average standard deviation of the average LFO (or VLFO) in block 1 and block 2.

$$\mathrm{coefcor}\;=\left(\;\frac{N-3}{\;N-2.25}\;\right)*\sqrt{\left(\;\frac{N-2}{N}\;\right)}$$

$${\mathrm{Cohen}}^{\prime }\;s\;d=\frac{\left(\left(\mu_{1}-\mu_{2}\right)*\mathrm{coefcor}\right)}{\mathrm{SD}}$$

$$\mathrm{SD}=\sqrt{\frac{\sigma_{1}^{2}+\sigma_{2}^{2}}{2}}$$

Cliff’s delta is a nonparametric effect size calculated with the following formula:

$${\mathrm{Cliff}}^{\prime }s\;\mathrm{delta}=\frac{1}{N^{2}}\underset{i,j}{\sum }\left[x_{1,j}>x_{2,j}\right]-[x_{1,j}<x_{2,j}]$$

Here, the summation covers the six patients, i.e., (i = 1 to 6, j = 1 to 6). x_1,j_ is the average LFO or VLFO power in one experimental block (e.g., m1) and x_2,j_ is the average LFO or VLFO power in the previous experimental block (e.g., b1). The “[ ]” denotes an Iverson bracket. This means that $\left[x_{1,j}>x_{2,j}\right]=1$ when $x_{1,j}>x_{2,j}$ and $\left[x_{1,j}<x_{2,j}\right]=0$ when $x_{1,j}<x_{2,j}$.

Cohen’s d and Cliff’s delta were calculated comparing the power within the LFO or VLFO range of frequency during the subsequent experimental blocks for each subject. Thus, the effect sizes of changes in power within LFO and VLFO among the subsequent pairs of experimental blocks were obtained (b1 to m1, m1 to b2, b2 to m2, and m2 to b3).

### 2.6. Statistical Tests

We performed group-level tests of the null hypothesis that the population mean of the LFO.Ratio for a given experimental block pair (e.g., b1 to m1) was equal to the subsequent block pair (in this case, m1 to b2). We performed separate tests for each subsequent block pair (e.g., b1–m1 vs. m1–b2) for LFO.Ratio, VLFO.Ratio, O2Hb, and HHb in both hemispheres. Since the power ratio values were paired in nature, we used the two-sided Wilcoxon signed rank test (significance threshold *p* < 0.05).

## 3. Results

### 3.1. Power Spectrum Analysis, a Qualitative Assessment

The qualitative analysis of the power spectra of the fNIRS signal in the six subjects confirmed a common pattern of distribution of power, with a peak under the frequency of 0.05 Hz (VLFO) and a second peak of power between 0.05 and 0.2 Hz (LFO). Focusing on the transition from the first baseline (b1) to the first music block (m1), we could identify a common pattern of power increase in the frequency region of LFO. This pattern was more evident for the left hemisphere.

**Figure 4:**
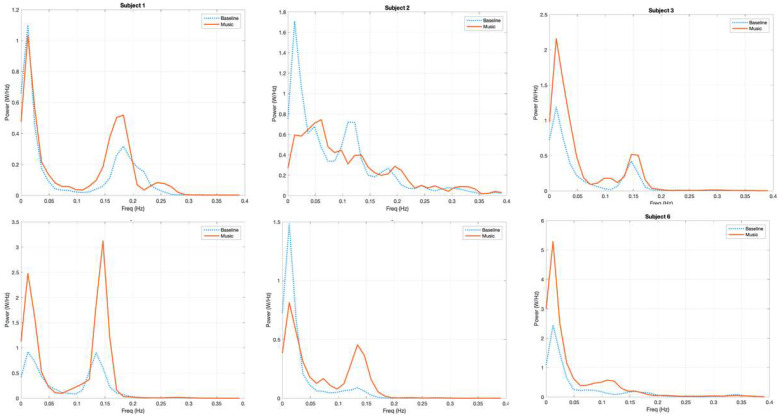
Power spectrum analysis of O2Hb fNIRS signal of each subject for the left hemisphere during the first baseline (blue, dashed curve) and the first music block (red, solid curve). The *y*-axis expresses the values of power (W/Hz); the *x*-axis shows the frequencies (Hz). Note that the values of power are variable for every subject due to different light conduction properties of the skin (e.g., depending on pigmentation and dryness) and optode adhesion.

**Figure 5:**
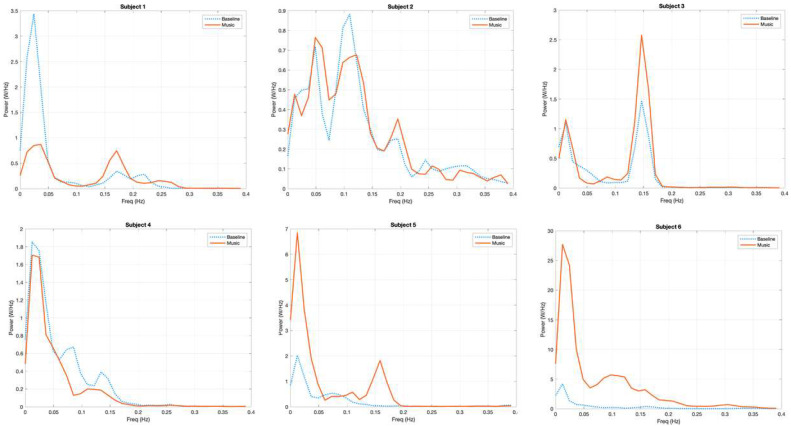
Power (W/Hz) spectrum analysis of O2Hb fNIRS signal of each subject for the right hemisphere during the first baseline (blue, dashed curve) and the first music block (red, solid curve). The *y*-axis expresses the values of power (W/Hz); the *x*-axis shows the frequencies (Hz). Note that the values of power are variable for every subject due to different light conduction properties of the skin (e.g., depending on pigmentation and dryness) and optode adhesion.

In Figure 4 (for the left hemisphere) and Figure 5 (for the right hemisphere), the power spectra of O2Hb for each subject during baseline and during the first music session are displayed. Considering the left hemisphere, a peak of spectral power was evident in the LFO region (here, 0.05–0.2 Hz); the power in this range of frequencies increased in subjects 1, 3, 4, 5, and 6 while listening to music. In addition, in the right hemisphere, a peak of spectral power was evident in the frequency range of 0.05–0.2 Hz; the power in this range of frequencies increased only in subjects 1, 3, 5, and 6 during m1 as compared to b1. We maintained the original values of power without normalizing them; as expected, there was great variability in the different subjects due to the different light conduction properties of the skin (e.g., depending on pigmentation and dryness) and optode adhesion.

### 3.2. Measuring the Response to Music, a Quantitative Approach

#### 3.2.1. LFO

**Figure 6:**
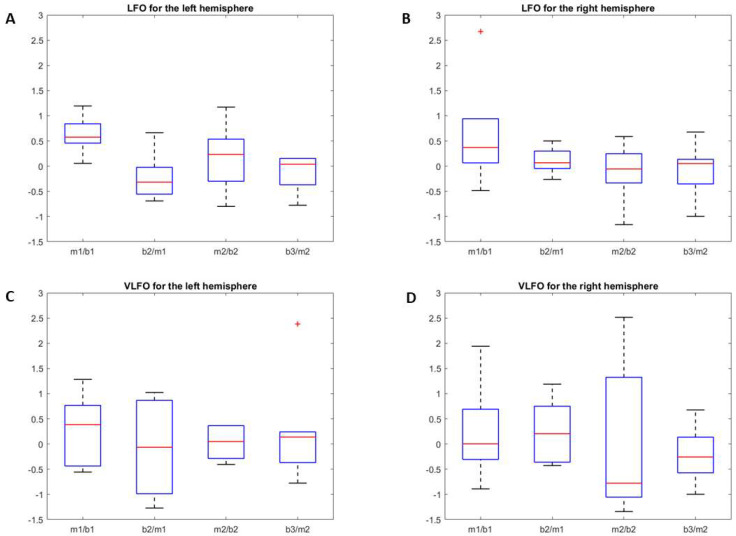
Boxplot showing, on the *y*-axis, the ratio of LFO power (low-frequency oscillation) and VLFO power (very-low-frequency oscillation) for O2HB. On the *x*-axis, the subsequent experimental blocks are shown: (m1/b1) from the first baseline to the first music period; (b2/m1) from the first music period to the second baseline; (b2/m2) from the second baseline to the second music block; (b3/m2) from the second music block to the third baseline. (**A**) Ratios of LFO power in the left hemisphere. After an increase in LFO power in m1/b1, a significant decrease is shown in b2/m1. (**B**) Ratios of LFO power in the right hemisphere. (**C**) Ratios of VLFO power in the left hemisphere. (**D**) Ratios of VLFO power in the right hemisphere.

The LFO.Ratio for O2Hb in the different experimental blocks is shown in a boxplot diagram in Figure 6A (left hemisphere) and 6B (right hemisphere). For the left hemisphere, the LFO.Ratio increases in the transition from b1 to m1 and decreases when music playback ends (m1 to b2). It increases again after the transition from b2 to m2. The values of LFO.Ratio for each pair of experimental blocks, along with outcomes of statistical tests of the LFO.Ratio between subsequent block pairs, are shown in Table 1. The effect sizes of changes in the average spectral power of LFO between each pair of experimental blocks are shown in Table 2.

##### O2Hb

For the left hemisphere, the mean power of LFO showed an increase from the first baseline to the first music block m1/b1 (LFO.Ratio mean = 0.617, SD = 0.384), and then it decreased from m1 to b2 (LFO.Ratio_b2/m1_ mean = −0.206, SD = 0.498). It rose again slightly from b2 to m2 m2/b2 (LFO.Ratio mean 0.179 SD 0.701) and to the b3 block b3/m2 (LFO.Ratio mean 0.516 SD 1.748). In the pairwise comparison between subsequent pairs of experimental blocks, the LFO.Ratio for m1/b1 was significantly greater than for b2/m1 (Wilcoxon signed rank test, rank statistic = 21, *p* = 0.031); moreover, the change in LFO power from b1 to m1 had a large effect size (Cohens’ d = 1; Cliff’s delta = 0.6). We did not find any other statistically significant transitions in the left hemisphere for O2Hb. Considering the right hemisphere, an increase in LFO (medium effect size) could be observed in m1/b1 (LFO.Ratio mean = 0.655, SD = 0.710); a small increase also occurred in b2/m1 (LFO.Ratio mean = 0.104, SD 0.270) and a decrease was observed in m2/b2 and b3/m2 (LFO.Ratio_m2/b2_ mean = −0.128, SD = 0.599, LFO.Ratio_b3/m2_ mean = −0.072, SD = 0.561).

**Figure A2:**
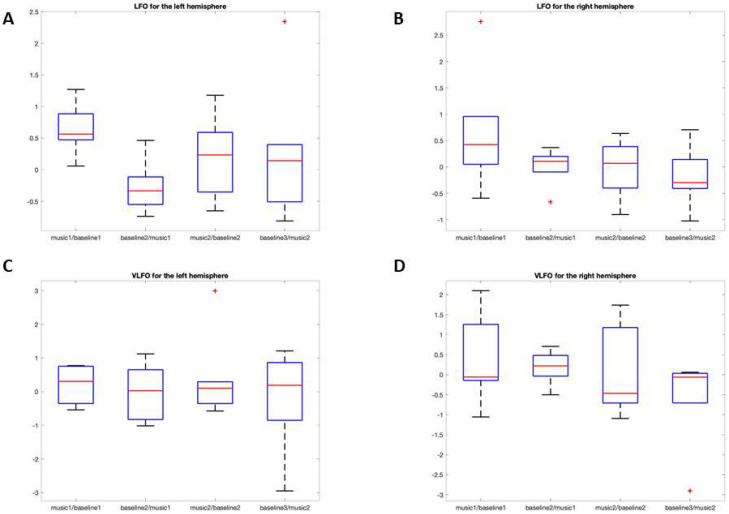
Boxplot showing, on the *y*–axis, the ratio of LFO power (low-frequency oscillation) and VLFO power (very-low-frequency oscillation) for O2HB calculated using an alternative cut–off of 0.05 Hz between LFO and VLFO; the same pattern of variations already observed with the cut–off of 0.04 Hz can be observed with this alternative cut–off. On the *x*–axis, the subsequent experimental blocks are shown: (m1/b1) from the first baseline to the first music period; (b2/m1) from the first music period to the second baseline; (b2/m2) from the second baseline to the second music block; (b3/m2) from the second music block to the third baseline. (**A**) Ratios of LFO power in the left hemisphere. After an increase in LFO power in m1/b1, a significant decrease is shown in b2/m1. (**B**) Ratios of LFO power in the right hemisphere. (**C**) Ratios of VLFO power in the left hemisphere. (**D**) Ratios of VLFO power in the right hemisphere.

**Figure A3:**
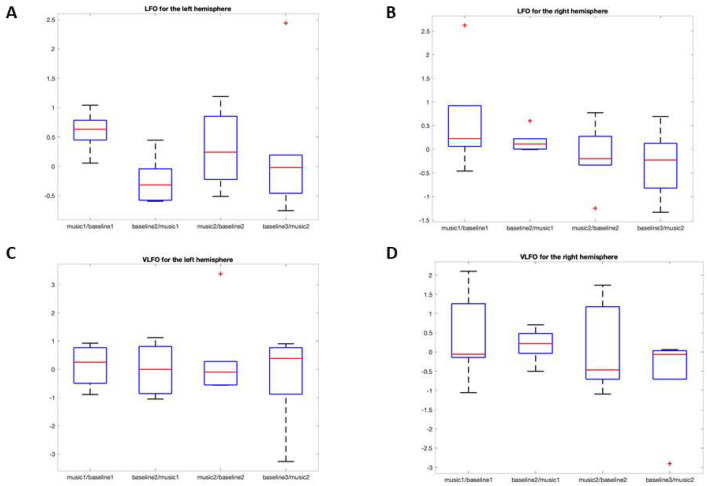
Boxplot showing, on the *y*-axis, the ratio of LFO power (low-frequency oscillation) and VLFO power (very-low-frequency oscillation) for O2HB calculated using an alternative cut-off of 0.03 Hz between LFO and VLFO; the same pattern of variations already observed with the cut-off of 0.04 Hz can be observed with this alternative cut-off. On the *x*-axis, the subsequent experimental blocks are shown: (m1/b1) from the first baseline to the first music period; (b2/m1) from the first music period to the second baseline; (b2/m2) from the second baseline to the second music block; (b3/m2) from the second music block to the third baseline. (**A**) Ratios of LFO power in the left hemisphere. After an increase in LFO power in m1/b1, a significant decrease is shown in b2/m1. (**B**) Ratios of LFO power in the right hemisphere. (**C**) Ratios of VLFO power in the left hemisphere. (**D**) Ratios of VLFO power in the right hemisphere.

**Figure A4:**
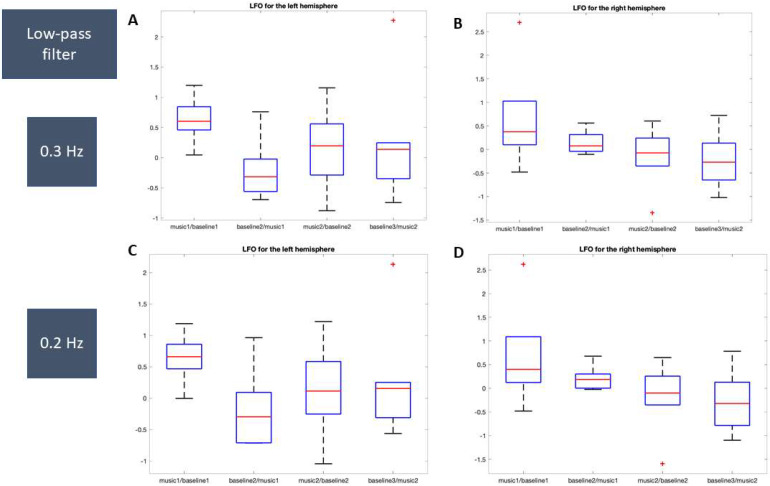
Boxplot showing, on the *y*-axis, the ratio of LFO power (low-frequency oscillation) for O2HB calculated using alternative low-pass filters (0.3 and 0.2 Hz) to identify the LFO spectral region. On the *x*-axis, the subsequent experimental blocks are shown: (m1/b1) from the first baseline to the first music period; (b2/m1) from the first music period to the second baseline; (b2/m2) from the second baseline to the second music block; (b3/m2) from the second music block to the third baseline. The same patterns of change previously observed with the original bandpass filter (0.4–0.04) are still observed. (**A**,**B**) Ratios of LFO power (here defined by a bandpass filter of 0.3–0.04 Hz) power in the left (**A**) hemisphere and (**B**) right hemisphere. (**C**,**D**) Ratios of LFO power (here defined by a bandpass filter of 0.2–0.04 Hz) in the right (**C**) and left (**D**) hemisphere.

No significant difference was found after pairwise comparison of LFO values between subsequent pairs of experimental blocks in the right hemisphere. The analyses were repeated adopting alternative values of cut-offs between LFO and VLFO, showing the same pattern found with the initial cut-off of 0.04 Hz (the results are shown in the Appendix A in Figure A2 and Figure A3). Alternative low-pass filters for LFO were also explored (Figure A4).

##### HHb

For the left hemisphere, the mean power of LFO increased in m1/b1 (LFO.Ratio mean = 0.267, SD = 0.301) and b2/m1 (LFO.Ratio mean = 0.670, SD = 1.110), followed by a slight decrease in m2/b2 (LFO.Ratio mean = −0.363, SD = 1.126), and then a slight increase in b3/m2 (LFO.Ratio mean = 0.432, SD = 1.605) (Table 2). No significant difference was found after pairwise comparison of LFO values between subsequent pairs of experimental blocks.

For the right hemisphere, an increase in LFO power was observed in m1/b1 (mean 0.583 SD 0.591), followed by a further slight increase in b2/m1 (LFO.Ratio mean 0.230 SD 0.695); a decrease in LFO power was then observed in m2/b2 (LFO.Ratio mean −0.500 SD 0.599) and b3/m2 (LFO.Ratio mean −0.110 SD 0.480). No significant difference was found after pairwise comparison of LFO values between subsequent experimental blocks.

#### 3.2.2. VLFO

The VLFO.Ratio for O2Hb in each pair of experimental blocks is displayed in Figure 6C (left hemisphere) and Figure 6D (right hemisphere), values of VLFO.Ratio for O2Hb and HHb are shown in the Table 3, and the effect sizes of changes in VLFO power are shown in Table 2. Differently to LFO, changes in VLFO power during the different experimental condition were markedly smaller and less consistent among the subjects. No significant differences could be found among the values of VLFO.Ratio.

##### O2Hb

For the left hemisphere, the mean power of VLFO showed an increase in m1/b1 (VLFO.Ratio mean = 0.304, SD = 0.708); it remained rather constant in b2/m1 (VLFO.Ratio mean = −0.081, SD = 1.029), and then an increase was observed in m2/b2 (VLFO.Ratio mean = 0.529, SD = 1.438) and in b3/m2 (VLFO.Ratio mean = 0.293, SD = 1.100). No significant difference was found after comparison of LFO values between subsequent pairs of experimental blocks. For the right hemisphere, an increase in VLFO power in m1/b1 could still be observed (mean = 0.240, SD = 0.981). VLFO power further increased in b2/m1 (mean = 0.261, SD = 0.648), and then it remained rather constant in m2/b2 (mean = −0.017, SD = 1.561) and it decreased in b3/m2 (mean = −0.212, SD = 0.579). No significant difference was found after comparison of VLFO values between subsequent pairs of experimental blocks. The analyses were repeated adopting alternative values of cut-offs between LFO and VLFO, showing the same pattern found with the initial cut-off of 0.04 Hz (the results are shown in Appendix A Figure A2 and Figure A3). Additionally, when adopting slightly different low-pass values for LFO, we could find the same patterns of change in LFO power.

##### HHb

For the left hemisphere, the mean power of VLFO showed a decrease in *m1/b1* (VLFO.Ratio mean = −0.255, SD = 0.916); then, it increased in b2/m1 (mean = 0.533, SD = 1.21), decreased in m2/b2 (mean = −0.287, SD = 1.429), and increased again in b3/m2 (mean = 0.507, SD = 1.787). No significant difference was found after pairwise comparison of VLFO values between subsequent experimental blocks. For the right hemisphere, first, a decrease in the mean VLFO power was observed in m1/b1 (VLFO.Ratio mean = −0.255, SD = 0.916). Then, the mean VLFO power increased in b2/m1 (mean = 0.613, SD = 0.837) and decreased again in m2/b2 (VLFO.Ratio mean = −0.563, SD = 0.915), while it was rather constant in b3/m2 (VLFO.Ratio mean = 0.110, SD = 0.480). No significant difference was found after pairwise comparison of VLFO values between subsequent experimental blocks.

#### 3.2.3. Systemic Vital Parameters

We measured the median value of cardiovascular parameters that might indirectly explain the change in O2Hb or HHb during the experiment. We tested whether the population mean value of a (median) cardiovascular parameter differed between two subsequent blocks. Results of group-level tests on median values: (n = 6 data points, 1 value per subject) are reported in Table 4. No significant differences were found for heart rate and SpO2 between b1 and m1. During music, the respiratory rate was 1.2 cycles per minute higher than during baseline. Even though the size of the difference was small, it was statistically significant (Wilcoxon signed rank test, statistic = 0.0, *p* value = 0.04, *p* value is not exact due to ties).

## 4. Discussion

The purpose of this pilot study was to investigate fNIRS as a tool to measure the physiological cerebral response to environmental stimuli, and to explore the potential of fNIRS frequency analysis in the detection of cerebral activation. Our results show that the LFO power increased in all subjects after the first exposure to music and decreased again in the subsequent resting phase. Changes in LFO power were more pronounced in the left hemisphere. The VLFO increased in at least four volunteers during the first music exposure, but its response was less consistent and showed a rather small effect size. Previous studies have shown that LFO amplitude in the prefrontal area is higher during wakefulness in comparison to sleep in healthy subjects [32]. An increase in LFO power for O2Hb is associated with the task-related activation of motor areas of the brain [33]. Hence, the increase in LFO power that we observed during music exposure may reflect an activation of the prefrontal cortex. We observed a left lateralization of the supposed response to music, consistent with previous reports showing activation of the left frontal cortex participating in music perception, probably by processing musical semantic memory [2,34,35]. O2Hb is generally considered more reliable than HHb for fNIRS studies because it has a higher amplitude of change and, thus, a higher signal to noise ratio [36]. Consistently, the increase observed in LFO power was more pronounced in the O2Hb signal. During the second music exposure, an increase in LFO power was only observed in the O2Hb signal for the left hemisphere in 3 out of 6 subjects, and the effect size of the increase was rather small. A repetition-related reduction in the neural hemodynamic response of the prefrontal areas has already been observed in several fMRI and fNIRS studies [37,38,39] and could explain this finding, considering that each subject listened always to the same musical piece, repeated in a loop. Our results showing an increase in LFO power with left lateralization after first music exposure and a repetition-related habituation effect strongly suggest that specific cerebral information is contained in the fNIRS signal. The main strength of this study is that we could identify a typical pattern of brain response to music and our methods require only two optodes on the forehead. Furthermore, our simple experimental design requires only a few minutes of listening to music and, thus, could be applied relatively easily to the real-world context of patients requiring neurocritical care. However, further work is needed to understand how much of the signal contained in fNIRS oscillations simply reflects a systemic physiological phenomenon as part of the same sympathetic autonomic reaction to music. Indeed, music is known to influence respiration, probably accelerating the breathing rate proportionally to the tempo though the activation of the sympathetic system [40]. We detected a slightly significant change in the respiration frequency from the resting phase to music exposure; therefore, we cannot exclude a systemic indirect influence of respiratory rate on the cerebral oscillations—for instance, through a reduction in carbon dioxide levels. Noticeably, several physiological parameters, such as respiratory rhythm, blood pressure, and heart period variability, are characterized by prevalent rhythms of oscillations, centered, respectively, at approximately 0.00, 0.12, and 0.27 Hz. The spectral power in each frequency domain varies under the influence of the neural vegetative system [41], thus presenting impressive similarities to the pattern of oscillation observed in the fNIRS. A further limitation of our study is that we only measured the activity of the prefrontal areas, and we did not co-register primary auditory areas. However, our aim was to test a potential bedside diagnostic tool that we can use in the future to evaluate the responsiveness of neurocritical patients. Placing the optodes on the forehead allows the measurement of the activity of prefrontal areas that are associated with auditory consciousness, probably through the activation of the fronto-parietal and fronto-temporal networks [2,42,43]. This optode setup has also important practical advantages because it is not impeded by the presence of hair; access to the forehead and the adhesion of the optodes would be much more practical than focusing on temporal areas in patients mostly lying on their back. As our study included only six subjects, the results need validation with a greater number of subjects; moreover, our work was primarily focused on the feasibility assessment of a methodology. Specific fNIRS features of cortical activation should be further investigated, comparing the findings obtained from healthy subjects with pathological conditions involving loss of auditory perception. Moreover, our results may not reflect a specific response to favorite music, as we did not compare the cortical response to auditory stimulation with other types of music or with noise. It was beyond the aim of this study to assess the specificity for preferred music of the observed cortical response. The main information that we would need to assess the level of consciousness of the patients would be cortical responsivity to any external stimuli; response to favorite music is among the most promising targets to evaluate responsiveness in patients with disorders of consciousness [44]. Further measurements should be performed intermodally with parallel electrophysiology by means of EEG, acoustic evoked potentials, and/or functional brain imaging with fMRI.

## 5. Conclusions

This is the first fNIRS study on the cortical hemodynamic response to favorite music using a frequency domain approach. Our results could identify a typical pattern of brain response to music; this is the first feasibility assessment for the development of a fNIRS-based diagnostic tool capable of detecting signs of cognitive activation in the brain and, potentially, capable of supporting the clinical evaluation of responsiveness in critical neurological patients with disorders of consciousness.

## Figures and Tables

**Table 1:** LFO.Ratio between subsequent pairs of experimental blocks, for O2Hb (top) and HHb (bottom), for the left hemisphere (left column) and the right hemisphere (right column), for each of the six subjects. Pairwise comparison between LFO.Ratio obtained in the subsequent couples of experimental blocks was performed with Wilcoxon signed rank test; *p* values are reported between each couple of columns. (m1/b1) from baseline to the first music block; (b2/m1) from the first music block to the second baseline (m2/b2) from the second baseline to the second music block; (b3/m2) from the second music block to the third baseline. Rs = rank statistic.

O2Hb								
	Left Hemisphere				Right Hemisphere			
	m1/b1	b2/m1	m2/b2	b3/m2	m1/b1	b2/m1	m2/b2	b3/m2
Subj 1	0.458	−0.556	1.172	−0.776	0.297	0.299	0.248	−0.352
Subj 2	0.056	−0.136	0.536	0.123	0.065	−0.007	−0.333	0.677
Subj 3	0.498	−0.495	−0.300	0.155	0.445	−0.046	−0.119	0.136
Subj 4	0.841	−0.690	−0.040	−0.370	−0.484	0.141	0.587	−0.997
Subj 5	1.194	0.665	−0.800	4.012	0.942	0.501	0.006	0.107
Subj 6	0.653	−0.024	0.508	−0.046	2.670	−0.262	−1.161	−0.002
Mean (SD)	0.617 (0.384)	−0.206 (0.498)	0.179 (0.701)	0.516 (1.748)	0.655 (1.09)	0.104 (0.270)	−0.128 (0.597)	−0.072 (0.561)
two-sided Wilcoxon signed rank test		m1/b1 − 2/m1, *p* = 0.031 rs = 21	b2/m1 − m2/b2, *p* = 0.313 rs = 5	b3/m2 − m2/b2, *p* = 0.844 rs = 12		m1/b1 − b2/m1, *p* = 0.438 rs = 15	b2/m1 − m2/b2, *p* = 0.219 rs = 17	b3/m2 − m2/b2, *p* = 0.844 rs = 9
**HHb**								
	**Left Hemisphere**				**Right Hemisphere**			
	**m1/b1**	**b2/m1**	**m2/b2**	**b3/m2**	**m1/b1**	**b2/m1**	**m2/b2**	**b3/m2**
Subj 1	0.421	−0.037	0.098	−0.32588	0.305	0.172	−0.085	−0.241
Subj 2	0.284	0.355	0.214	0.006273	0.110	0.050	-0.556	0.666
Subj 3	0.702	0.132	−0.594	0.20135	0.876	−0.442	−0.334	0.180
Subj 4	−0.108	−0.054	0.799	−0.63941	−0.015	0.079	0.267	−0.290
Subj 5	0.344	2.850	−2.46	3.654578	0.645	1.581	−1.431	0.695
Subj 6	−0.039	0.777	−0.242	−0.30515	1.581	−0.054	−0.864	−0.351
Mean (SD)	0.267 (0.301)	0.670 (1.110)	−0.363 (1.126)	0.432 (1.605)	0.583 (0.591)	0.230 (0.695)	−0.500 (0.599)	−0.110 (0.480)
two-sided Wilcoxon signed rank test		m1/b1 − b2/m1, *p* = 0.562 rs = 7	b2/m1 − m2/b2, *p* = 0.313 rs = 16	b3/m2 − m2/b2, *p* = 1 rs = 11		m1/b1 − b2/m1, *p* = 0.437 rs = 15	b2/m1 − m2/b2, *p* = 0.156 rs = 18	b3/m2 − m2/b2, *p* = 0.313 rs = 5

**Table 2:** Effect size measures (Cohen’s d and Cliff’s delta) for LFO (top) and VLFO (bottom) for oxyhemoglobin (O2Hb, left) and deoxyhemoglobin (HHb, right).

LFO								
O2Hb					HHb			
	left		right		left		right	
	Cohen’s d	Cliff’s delta	Cohen’s d	Cliff’s delta	Cohen’s d	Cliff’s delta	Cohen’s d	Cliff’s delta
m1 vs. b1	1.0	0.6	0.4	0.4	0.6	0.2	0.6	0.4
b2 vs. m1	−0.3	−0.3	0.3	0.0	0.4	0.4	0.2	0.2
m2 vs. b2	0.2	0.2	−0.1	−0.1	−0.2	−0.2	−0.5	−0.4
b3 vs. m2	0.2	0.3	−0.2	−0.2	0.2	0.2	0.1	0.1
**VLFO**								
O2Hb					HHb			
	left		right		left		right	
	Cohen’s d	Cliff’s delta	Cohen’s d	Cliff’s delta	Cohen’s d	Cliff’s delta	Cohen’s d	Cliff’s delta
m1 vs. b1	0.3	0.2	0.2	0.0	−0.2	0.0	−0.2	−0.1
b2 vs. m1	−0.1	−0.1	0.3	0.2	0.3	0.2	0.5	0.3
m2 vs. b2	0.2	0.2	0.0	0.0	−0.1	−0.3	−0.4	−0.2
b3 vs. m2	−0.1	0.0	−0.3	−0.3	0.3	0.4	0.0	0.0

**Table 3:** VLFO.Ratio (low-frequency oscillation) between the subsequent pairs of experimental blocks, for O2Hb (top) and HHb (bottom), for the left hemisphere (left column) and the right hemisphere (right column). Pairwise comparison between VLFO.Ratio obtained in subsequent pairs of experimental blocks was performed with Wilcoxon signed rank test. (m1/b1) from baseline to the first music block; (b2/m1) from the first music block to the second baseline (m2/b2) from the second baseline to the second music block; (b3/m2) from the second music block to the third baseline. Rs = rank statistic.

O2Hb								
	Left Hemisphere				Right Hemisphere			
	m1/b1	b2/m1	m2/b2	b3/m2	m1/b1	b2/m1	m2/b2	b3/m2
Subj 1	0.255	−1.27	0.365	−0.776	−0.890	−0.427	1.323	−0.352
Subj 2	−0.557	0.627	−0.287	0.123	0.171	1.189	−1.054	0.677
Subj 3	0.766	−0.987	−0.405	0.155	−0.162	0.467	-0.853	0.136
Subj 4	1.283	−0.755	3.411	−0.370	−0.305	−0.360	2.516	−0.997
Subj 5	−0.436	1.023	−0.012	2.386	0.691	−0.054	−0.700	−0.162
Subj 6	0.518	0.867	0.106	0.241	1.94	0.751	−1.34	−0.571
Mean (SD)	0.304 (0.708)	−0.081 (1.029)	0.529 (1.438)	0.293 (1.100)	0.240 (0.981)	0.261 (0.648)	−0.017 (1.561)	−0.212 (0.579)
two-sided Wilcoxon signed rank test		m1/b1 − 2/m1, *p* = 0.437 rs = 15	b2/m1 − m2/b2, *p* = 0.844 rs = 9	b3/m2 − m2/b2, *p* = 1 rs = 10		m1/b1 − b2/m1, *p* = 1 rs = 11	b2/m1 − m2/b2, *p* = 0.844 rs = 12	b3/m2 − m2/b2, *p* = 1 rs = 10
**HHb**								
	**Left Hemisphere**				**Right Hemisphere**			
	**m1/b1**	**b2/m1**	**m2/b2**	**b3/m2**	**m1/b1**	**b2/m1**	**m2/b2**	**b3/m2**
Subj 1	0.283	−0.377	0.251	−0.326	0.616	−0.521	0.300	−0.241
Subj 2	−0.789	1.56	−0.661	0.006	−1.071	1.962	−1.627	0.666
Subj 3	−0.247	0.648	−0.685	0.201	−1.223	0.359	−1.296	0.180
Subj 4	−0.913	0.148	1.94	-0.639	−0.332	0.802	0.732	−0.290
Subj 5	−0.866	2.225	−2.445	4.106	−0.579	0.917	−0.861	0.695
Subj 6	0.903	−1.004	−0.118	−0.305	1.059	0.162	−0.629	−0.351
Mean (SD)	−0.271 (0.738)	−0.533 (1.21)	−0.287 (1.429)	0.507 (1.787)	−0.255 (0.916)	0.613 (0.837)	−0.563 (0.915)	0.110 (0.480)
two-sided Wilcoxon signed rank test		m1/b1 − b2/m1, *p* = 0.312 rs = 5	b2/m1 − m2/b2, *p* = 0.562 rs = 14	b3/m2 − m2/b2, *p* = 0.687 rs = 8		m1/b1 − b2/m1, *p* = 0.218 rs = 4	b2/m1 − m2/b2, *p* = 0.156 rs = 18	b3/m2 − m2/b2, *p* = 0.312 rs = 5

**Table 4:** Wilcoxon signed rank test for median values of heart rate, SpO2, and respiratory rate for three experimental blocks: baseline 1 (b1), music 1 (m1), and baseline 2 (b2). rs = rank statistic.

	b1 vs. m1	m1 vs. b2	b1 vs. b2
Heart rate	rs = 8.5, *p* = 0.84375	rs = 5, *p* = 0.3125	rs = 1, *p* = 0.61
SpO2	rs = 9.5, *p* = 1	rs = 1, *p* = 0.65	rs = 5, *p* = 0.3125
Respiratory rate	rs = 0.0, *p* = 0.04	rs = 1.5, *p* = 0.1974	rs =2, *p* = 0.5637

## Data Availability

All our data are available upon request.
